# Minimally Invasive Hematoma Evacuation for Acute Spontaneous Supratentorial Intracerebral Hemorrhage: A Systematic Review and Meta‐Analysis With Trial Sequential Analysis

**DOI:** 10.1002/brb3.71815

**Published:** 2026-10-14

**Authors:** Sahar Saleem, Zaryab Bacha, Khushal Khan, Khawaja Arham Jawaid, Muhammad Abbas, Shahab Iqbal, Umer Zaryab Khan, Muhammad Waqas, Saja Mohammed Taher Al‐Zamili, Humaira Obaid, Zuha Kashif, Abdullah Abdullah, Zahra Rehman, Javeria Javed, Kamil Ahmad Kamil

**Affiliations:** ^1^ Allama Iqbal Medical College Lahore Pakistan; ^2^ Khyber Medical College Peshawar Pakistan; ^3^ Sharif Medical City Hospital Lahore Pakistan; ^4^ Ayub Medical College Abbottabad Pakistan; ^5^ Gomal Medical Dera Ismail Khan Pakistan; ^6^ College of Medicine Al‐Ayen University Thi‐Qar Iraq; ^7^ Jinnah Sindh Medical University Karachi Pakistan; ^8^ Chief, Internal Medicine Department Nasrat Bashir Curative Hospital Kandahar Afghanistan

**Keywords:** ICH, intracerebral hemorrhage, meta‐analysis, minimally invasive hematoma evacuation, mortality, randomized controlled trials, surgical intervention

## Abstract

**Background:**

Intracerebral hemorrhage (ICH) is a severe form of stroke associated with high morbidity and mortality. While surgical evacuation aims to reduce hematoma burden and improve outcomes, its clinical benefit over medical management remains uncertain. Previous systematic reviews have evaluated a broad range of surgical interventions for ICH; however, evidence specific to MIS remains less clearly defined. Therefore, we performed a systematic review and meta‐analysis to assess the efficacy and safety of minimally invasive hematoma evacuation in patients with acute spontaneous supratentorial ICH.

**Methods:**

This meta‐analysis was performed in accordance with PRISMA 2020 guidelines and Cochrane recommendations. A comprehensive search of MEDLINE, Embase, Cochrane CENTRAL, and ClinicalTrials.gov was conducted from inception to February 2026. We included randomized controlled trials that compared minimally invasive hematoma evacuation with medical or conventional surgical management. Risk ratios (RR) and mean differences (MD) were pooled using a random‐effects model.

**Results:**

A total of 12 RCTs involving over 2302 patients were included. Minimally invasive hematoma evacuation significantly reduced all‐cause mortality (RR = 0.77, 95% CI 0.61–0.97; *p* = 0.02) and 6‐month mortality (RR = 0.79, 95% CI 0.64–0.96; *p* = 0.02). No significant differences were observed in 7‐day or 30‐day mortality, although a trend favoring the intervention was noted. Functional outcomes, including mRS 0–1 and mRS 0–3, did not improve significantly. Among secondary outcomes, minimally invasive approaches were associated with greater hematoma volume reduction (MD = 62.47; *p* = 0.04) but also a higher risk of rebleeding (RR = 2.37; *p* = 0.002) and cardiac events (RR = 5.08; *p* = 0.009). No significant differences were found in ischemic stroke, renal failure, gastrointestinal bleeding, seizures, urinary tract infections, or length of hospital and ICU stay.

**Conclusions:**

Minimally invasive hematoma evacuation significantly decreases mortality in patients with spontaneous ICH, especially with endoscopic techniques; however, it does not markedly enhance functional outcomes and may elevate the risk of complications such as rebleeding and cardiac events. These results underscore the necessity for meticulous patient selection and additional high‐caliber trials to enhance treatment strategies.

## Graphical abstract

Acute spontaneous supratentorial ICH is associated with high morbidity and mortality. In 12 RCTs involving > 2302 patients, we compared minimally invasive hematoma evacuation (MIS) with medical management or conventional surgical evacuation. MIS reduced all‐cause and 6‐month mortality and achieved greater hematoma volume reduction, but did not significantly improve functional outcomes. MIS was associated with increased risks of rebleeding and cardiac events, with no significant differences in other clinical outcomes.

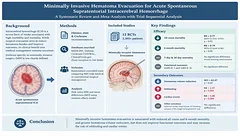

## 1 Introduction

Intracerebral haemorrhage (ICH) is a life‐threatening neurological condition defined by spontaneous bleeding within the brain parenchyma. The most common etiologies of spontaneous ICH are hypertensive vasculopathy and cerebral amyloid angiopathy, whereas secondary causes include vascular malformations, intracranial tumours, and hemorrhagic transformation following cerebral thrombosis (Phung et al. 2025). It accounts for 10% to 15% of all strokes and is associated with the highest morbidity and mortality rates of any stroke subtype, with early mortality estimated as high as 30% to 40% (Greenberg et al. 2022; Ziai et al. 2024). This high mortality rate is largely attributable to early limitations in care based on a perceived poor prognosis and is commonly associated with admission severity factors, including hematoma volume, intraventricular extension, haemorrhage location, patient age, and Glasgow Coma Scale (GCS) score (De Rosa et al. 2023). In 2019, ICH accounted for approximately 2.89 million deaths globally and represented 27.9% of all incident stroke cases, with a rising burden observed in low‐ and middle‐income countries (Feigin et al. 2021). The major risk factors underlying this burden are elevated blood pressure (BP; accounting for 57.9% of ICH disability‐adjusted life years), followed by increased body mass index (43.7%) and high plasma glucose levels (34.8%) (Feigin et al. 2021).

Current management of acute spontaneous ICH comprises both medical and surgical approaches. Early surgical intervention is intended to decrease hematoma volume, reduce intracranial pressure (ICP), and limit neurological deterioration. Surgical management of spontaneous supratentorial ICH includes decompressive craniectomy (DC), conventional craniotomy (CC), and minimally invasive approaches (MIS), which comprise endoscopic evacuation, stereotactic aspiration with thrombolysis (urokinase or rt‐PA), craniopuncture aspiration, early minimally invasive trans‐sulcal parafascicular surgery, and stereotactic‐guided clot removal. Medical management, on the other hand, focuses on controlling blood pressure and ICP and fixing coagulopathies to stabilize the patient clinically (Al‐Kawaz et al. 2020). Both strategies offer distinct advantages and limitations, which call for careful comparative evaluation to identify the most effective treatment approach.

Numerous studies have examined the outcomes of these interventions, reflecting the nuanced benefits and limitations of surgical and medical approaches. Arthur et al. suggested that MIS using the Artemis Neuro Evacuation Device did not improve 180‐day disability or 30‐day mortality compared with standard medical management but showed good technical performance and improved 30‐day outcomes in both deep and lobar ICH (Arthur et al. 2025). Kim et al. found that in patients with hematoma volumes < 30 cm^3^ and GCS ≥ 13 but with moderate to severe motor deficits limiting independence, stereotactic‐guided evacuation can improve functional outcomes and restore independence in daily activities (Kim and Kim 2009). According to Akram M. J. et al., surgical treatment did not result in significantly different outcomes compared with conservative medical management in patients with lobar ICH (Akram et al. 2022). Wang et al. reported that MIS resulted in better functional outcomes than conservative medical management in patients with ICH (Wang et al. 2009). A study by Zhang et al. shows that while surgical intervention can reduce mortality, it may negatively impact functional independence. Minimally invasive surgery may offer benefits for patients with large hematomas (30–100 mL) (Zhang et al. 2025).

Given the expanding evidence base and variability among individual studies, a thorough synthesis of available data is necessary. Therefore, we conducted a systematic review and meta‐analysis of studies evaluating minimally invasive hematoma evacuation for acute spontaneous supratentorial ICH, compared with medical management and conventional craniotomy.

## 2 Methods

### 2.1 Study Design and Protocol Registration

This meta‐analysis was conducted following the Cochrane Handbook for Systematic Reviews of Interventions and reported in accordance with the PRISMA 2020 guidelines (Higgins et al. 2024). The study protocol has been registered and is available on PROSPERO under the ID CRD420261352217

### 2.2 Data Resources and Search Strategy

Cochrane Central Register of Controlled Trials, PubMed, MEDLINE, Embase, and ClinicalTrials.gov were used to perform a thorough electronic search from their inception to February 2026. To capture all relevant studies, we performed both backward snowballing (reviewing reference lists) and forward snowballing (tracking citations) of the included articles. The reference lists of the included studies and related systematic reviews were also examined to identify additional relevant studies. The following terms, “intracerebral hemorrhage (ICH),” “hematoma, intracranial,” “supratentorial hemorrhage,” “minimally invasive surgical procedures,” “hematoma evacuation,” “endoscopy,” “stereotaxic techniques,” “thrombolytic therapy,” and “tissue plasminogen activator,” were used as either Medical Subject Headings terms or keywords.

### 2.3 Study Selection and Eligibility Criteria

All identified articles were imported into Rayyan AI for deduplication. Two independent reviewers screened the titles and abstracts of the remaining studies. Full‐text assessment was conducted for articles deemed potentially eligible based on predefined inclusion criteria. Disagreements were resolved through discussion with a third reviewer.

Studies were included if they met the following criteria: (Phung et al. 2025) population: adults (18–80 years) with acute spontaneous supratentorial (ICH) having a hematoma volume of 20–80 mL; a hematoma volume range of 20–80 mL was considered representative of the patient population most commonly targeted for minimally invasive hematoma evacuation, as hematomas smaller than 20 mL are often managed conservatively because of their relatively favorable prognosis, whereas hematomas larger than 80 mL are associated with extensive neurological injury and poor outcomes regardless of intervention. However, studies were not excluded solely on the basis of hematoma volume, and trials enrolling patients with volumes outside this range (e.g., 10–40 mL) were included if they satisfied all other eligibility criteria, symptom onset to randomization: within 24–72 h (Greenberg et al. 2022). Intervention: minimally invasive hematoma evacuation, including endoscopic evacuation, stereotactic aspiration with thrombolysis (urokinase or rt‐PA), craniopuncture aspiration, early minimally invasive trans‐sulcal parafascicular surgery, and stereotactic‐guided clot removal (Ziai et al. 2024). Control: guideline‐based best medical management alone (De Rosa et al. 2023). Study design: multicenter, randomized, controlled, open‐label trials comparing minimally invasive hematoma evacuation with best medical management (Feigin et al. 2021). Outcomes: studies were eligible if they reported at least one of the prespecified clinical outcomes: functional status at 90–180 days (mRS, Barthel Index, or utility‐weighted mRS); mortality at 30 and 180 days; hematoma volume reduction on CT; quality of life measures; or safety, including perioperative rebleeding, infection, or neurological deterioration. Key details of the included studies, including duration, inclusion and exclusion criteria, intervention details, primary outcomes, and results, are summarized in Table 1.

**TABLE 1:** Study characteristics.

Study ID	Country	Type of study	intervention	Control	Sample Size(n)		Male (%)	
					Intervention	Control	Intervention	Control
Wang 2009	China	RCT	Craniopuncture	Conservative	195	182	58	67
AUER 1989	Austria	RCT	Endoscopy	Medical group	50	50	56	66
Zuccarello 2025	Multicenter	Randomized feasibility study	Surgical group	Medical group	9	11	NR	NR
Teernstra 2002	Netherlands	RCT	Surgical group	Non surgical group	36	35	NR	NR
Vespa 2016	USA	RCT	Surgical group	Medical group	14	4	NR	NR
Hanley 2016	Multicenter	RCT	Minimally invasive surgery + recombinant tissue plasminogen activator	Medical care group	54	42	64	66
Kim 2009	Korea	RCT	Stereotactic‐guided hematoma evacuation	Conservative (medical) treatment	204	183	76	72
Arthur 2025	Multicenter	RCT	Minimally invasive surgery with Artemis + medical management	Medical management alone	154	82	64	61
HATTORI 2004	Japan	RCT	Stereotactic evacuation of the hematoma	Conservative treatment	121	121	71	77
Pradilla 2024	US	RCT	Surgery group	Control group	150	150	48	52

Abbreviations: NR: not reported, RCT: randomized controlled trail.

### 2.4 Exclusion Criteria

Studies were excluded if they were non‐randomized controlled trials or observational studies, including case reports, review articles, and cohort studies. Additionally, studies that did not report the outcomes of interest were excluded. Trials were also excluded if they provided insufficient data or did not meet the predefined inclusion criteria, such as those enrolling patients younger than 18 years. with a GCS score less than 5, or with hematoma volumes outside the 20–80 mL range.

### 2.5 Data Extraction

Data were independently extracted by two authors (KK and SS) using a preformatted Excel spreadsheet, with any discrepancies resolved by a third author (ZB). Raw data were calculated using percentages when needed, and relative information was collected from the text, tables, and figures of the included studies. Baseline characteristics included age, gender, and comorbidities such as hypertension, diabetes, and cardiovascular disease.

### 2.6 Outcomes

The primary outcome was all‐cause mortality, including overall mortality and mortality assessed at specific time points (7 days, 30 days, and 6 months). Secondary outcomes included functional outcomes measured using the modified Rankin Scale (mRS) (0–1 and 0–3), mean mRS score at 180 days, hematoma volume reduction on computed tomography (CT), and safety outcomes. For functional outcome assessment, mRS data were extracted according to the definitions reported in the original studies and pooled separately based on the thresholds provided by each trial. No new cut‐offs were derived, and studies reporting different mRS definitions were analyzed separately. Safety endpoints comprised rebleeding, cardiac events, ischemic stroke, renal failure, gastrointestinal bleeding, seizures, urinary tract infections, and length of hospital and intensive care unit stay.

### 2.7 Quality Assessment

The risk of bias (RoB) in the included randomized controlled trials (RCTs) was evaluated using the revised Cochrane RoB tool (RoB 2.0) (Sterne et al. 2019). This tool looks at bias in five areas: (Phung et al. 2025) bias that comes from the randomisation process, (Greenberg et al. 2022) bias that comes from not following the planned interventions, (Ziai et al. 2024) bias that comes from missing outcome data, (De Rosa et al. 2023) bias that comes from measuring outcomes, and (Feigin et al. 2021) bias that comes from choosing which results to report. Two investigators (K.K. and KAJ) independently assessed the RoB for each included study as high, low, or with some concerns of bias. The third author (Z.B.) resolved any disagreements regarding the bias.

### 2.8 Grade Assessment

The certainty of evidence in this meta‐analysis was evaluated using the Grading of Recommendations, Assessment, Development, and Evaluation (GRADE) framework, implemented via the GRADEpro Guideline Development Tool. (Santesso et al. 2016) Two independent reviewers (M.A. and U.Z.K.) assessed the evidence and classified it as high, moderate, low, or very low certainty. (Shao et al. 2023) Discrepancies were resolved through discussion and consensus.

### 2.9 Date Analysis

Statistical analyses were performed in RevMan 5.4. Binary outcomes were summarized using pooled risk ratios (RR) with 95% confidence intervals (CI). The Cochran Q test and *I* ^2^ statistic were used to check for heterogeneity. A *p* value of less than 0.10 and an *I* ^2^ value of more than 50% meant that there was substantial heterogeneity (Higgins et al. 2003). The DerSimonian and Laird random‐effects model was used for all analyses (DerSimonian and Laird 1986), and *P* < 0.05 was considered significant. Robustness was evaluated through leave‐one‐out sensitivity analysis, and trial sequential analysis (TSA) was conducted to assess the conclusiveness of pooled evidence for selected outcomes.

### 2.10 Trial Sequential Analysis (TSA)

TSA was conducted for the primary outcomes to evaluate the robustness of the findings with respect to type I and type II errors (Wetterslev et al. 2017). The required information size (RIS) was calculated assuming a type I error rate of 5% and a statistical power of 80%. Z‐score monitoring boundaries were used to assess evidence of benefit, harm, or futility of minimally invasive surgery compared to control in patients with acute spontaneous supratentorial ICH (Wetterslev et al. 2008).

## 3 Results

### 3.1 Search Results

A total of 6913 records were identified through database searching, including 1732 from MEDLINE (PubMed), 4911 from Embase, and 288 from the Cochrane CENTRAL database. After removal of 2104 duplicates, 4827 records remained for title and abstract screening. Of these, 4764 records were excluded due to their irrelevance to the study question. Sixty‐three full‐text articles were sought for retrieval, of which 4 could not be accessed. The remaining 59 articles underwent full‐text assessment for eligibility. Following detailed evaluation, 47 studies were excluded for the following reasons: wrong study design (*n* = 13), inappropriate population (*n* = 5), absence of a comparator group (*n* = 9), insufficient outcome data (*n* = 6), and publication type such as reviews, editorials, or case reports (*n* = 14). Ultimately, 12 randomized controlled trials (RCTs) met the inclusion criteria and were included in the qualitative and quantitative synthesis. As shown in Figure 1, the PRISMA flow diagram summarizes the study selection process.

**FIGURE 1:**
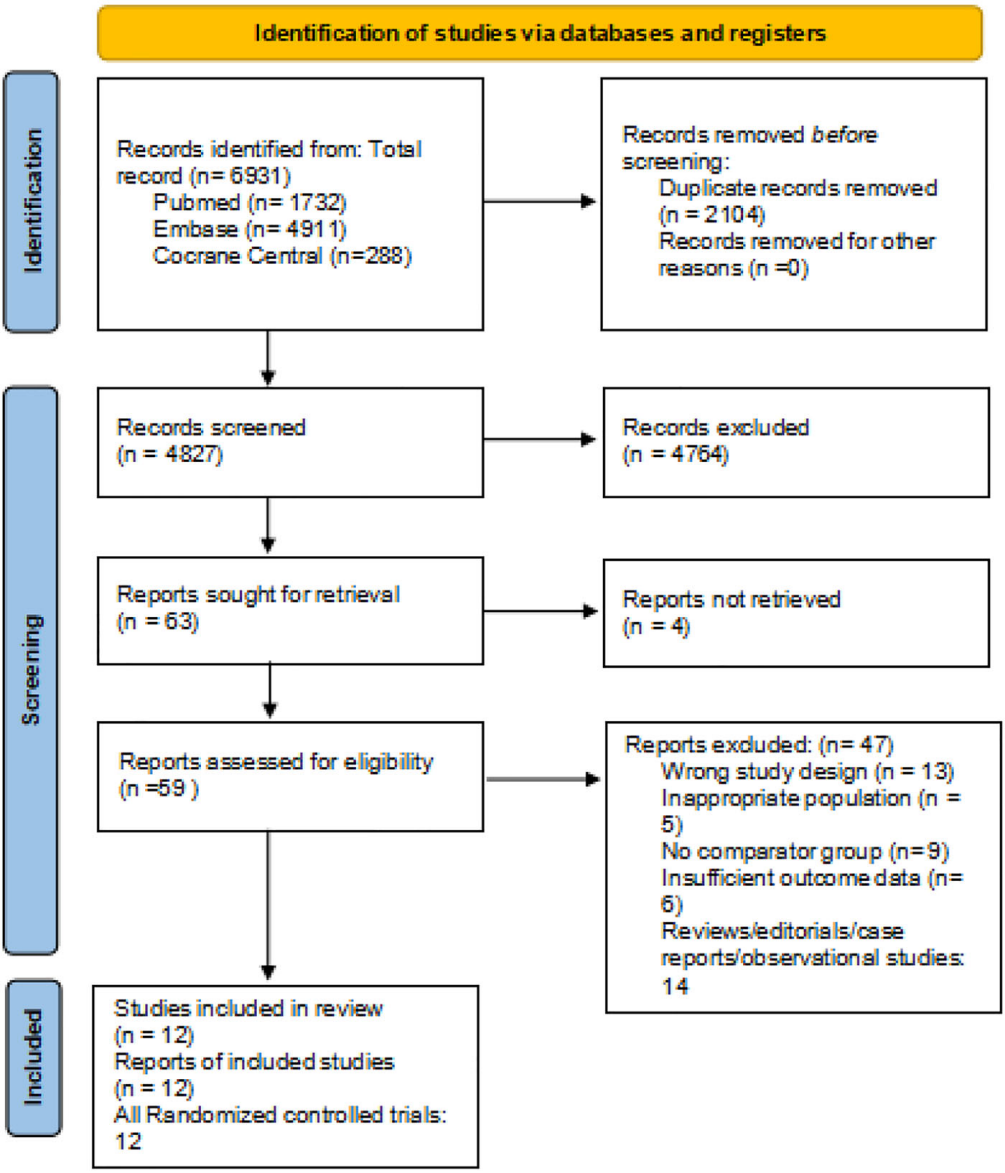
**PRISMA 2020 flow diagram**.

### 3.2 Study and Baseline Characteristics

The analysis included a total of 12 RCTs (Zuccarello et al. 1999; Teernstra et al. 2003; Pradilla et al. 2024; Hanley et al. 2019; Hattori et al. 2004; Miller et al. 2008; Wang et al. 2009; Kim and Kim 2009; Vespa et al. 2016; Arthur et al. 2025; Auer et al. 1989; Deng et al. 2022) with 2302 participants (1221 in the intervention group and 1081 in the control group). The studies were conducted across multiple regions, including Asia, Europe, and North America, with several multicenter trials, and they were published between 1989 and 2025.

All trials compared surgical or minimally invasive hematoma evacuation techniques—such as craniopuncture, endoscopic evacuation, stereotactic‐guided procedures, and minimally invasive surgery with adjuncts (e.g., recombinant tissue plasminogen activator or device‐assisted evacuation) against conservative or standard medical management. Sample sizes varied widely, ranging from small feasibility studies (*n* = 20) to larger multicenter trials (*n* > 200).

Baseline characteristics were generally comparable between intervention and control groups across the included trials. Mean age, reported in four studies, ranged from 56.6 to 64.0 years in the intervention groups and from 56.9 to 62.0 years in the control groups. The proportion of male participants varied between 48% and 76% in the intervention groups and 52% and 77% in the control groups. Baseline hematoma volume was reported in seven studies and ranged from 22.8 to 55.0 mL in the intervention groups and from 20.4 to 56.0 mL in the control groups. Hematoma location, when reported, included both lobar and deep hemorrhages, with deep hematomas accounting for 32%–68.2% of cases in the intervention groups and 29%–72% in the control groups. Overall, no major imbalances in demographic or clinical characteristics were observed between treatment arms within individual studies (Tables 1 and 2).

**TABLE 2:** Patient characteristics.

**Study ID**	**Age mean (SD)**		**Male, %**		**Hematoma volume (mL) mean ± SD**		**Hematoma location, %**			
							**Lobar**		**Deep**	
	**Intervention**	**Control**	**Intervention**	**Control**	**Intervention**	**Control**	**Intervention**	**Control**	**Intervention**	**Control**
Wang 2009	56.6 ± 9.7	56.9 ± 9.2	58	67	33.8 ± 5.2	31.3± 5	NR	NR	100	100
AUER 1989	NR	NR	56	66	NR	NR	NR	NR	NR	NR
Zuccarello 2025	NR	NR	NR	NR	39.4 ± 38.4	33.3 ± 26.2	NR	NR	NR	NR
Teernstra 2002	NR	NR	NR	NR	NR	NR	67	41	NR	NR
Vespa 2016	NR	NR	NR	NR	NR	NR	NR		NR	
Hanley 2016	60.7 ± 11	61.6 ± 12	64	66	48.2 ± 19.6	43.1 ± 15.3	33.3	35.7	66.7	64.3
Kim 2009	NR	NR	76	72	22.8 ± 11.6	20.4 ± 14.6	NR	NR	NR	NR
Arthur 2025	60 ± 12.6	60 ± 16.3	64	61	41.7 ± 18.7	40.4 ± 18.8	31.8	28	68.2	72
HATTORI 2004	NR	NR	71	77	48 ± 15.6	40 ± 12.3	NR	NR	NR	NR
Pradilla 2024	**64** ± 11.85	62 ± 16.3	48	52	55 ± 24.7	56 ± 24.7	68	71	32	29

### 3.3 Grade Assessment

The overall certainty of evidence ranged from high to low across outcomes. High‐quality evidence was observed for key endpoints, including all‐cause mortality, 30‐day and 6‐month mortality, rebleeding, cardiac events, and pneumonia, indicating strong confidence in these estimates. Moderate‐quality evidence was found for outcomes such as 7‐day mortality, functional outcomes (mRS 0–1 and 0–3), myocardial infarction, ischemic stroke, gastrointestinal bleeding, renal failure, and urinary tract infections, primarily downgraded due to imprecision. In contrast, low‐quality evidence was noted for mRS 0–6, symptomatic bleeding after 72 h, length of hospital and ICU stay, and serious adverse events, mainly due to inconsistency and imprecision. Overall, the and indirectness were not serious across studies, while imprecision and heterogeneity were the main factors contributing to the downgrading of evidence certainty Table S1.

### 3.4 Quality Assessment

Overall, the included studies demonstrated a predominantly low RoB. Most domains, including deviations from interventions, missing data, outcome measurement, and selective reporting, were rated as low risk across trials. Some concerns were noted in the randomization process in a few studies, leading to an overall rating of “some concerns” for those trials. Importantly, no study was judged to have a high RoB, indicating generally reliable and methodologically sound evidence Figure S1.

### 3.5 Meta‐analysis of Primary Outcomes

#### 3.5.1 All‐Cause Mortality

10 studies, including 2118 patients (1125 minimally invasive hematoma evacuation vs 993 control). The pooled random effect model demonstrated a statistically significant difference in all‐cause mortality for minimally invasive hematoma evacuation over control with a pooled risk ratio (RR = 0.77, 95% CI 0.61 to 0.97; *Z* = 2.25, *p* = 0.02). No heterogeneity was observed. As shown in Figure 2, minimally invasive hematoma evacuation was associated with a significant survival benefit.

**FIGURE 2:**
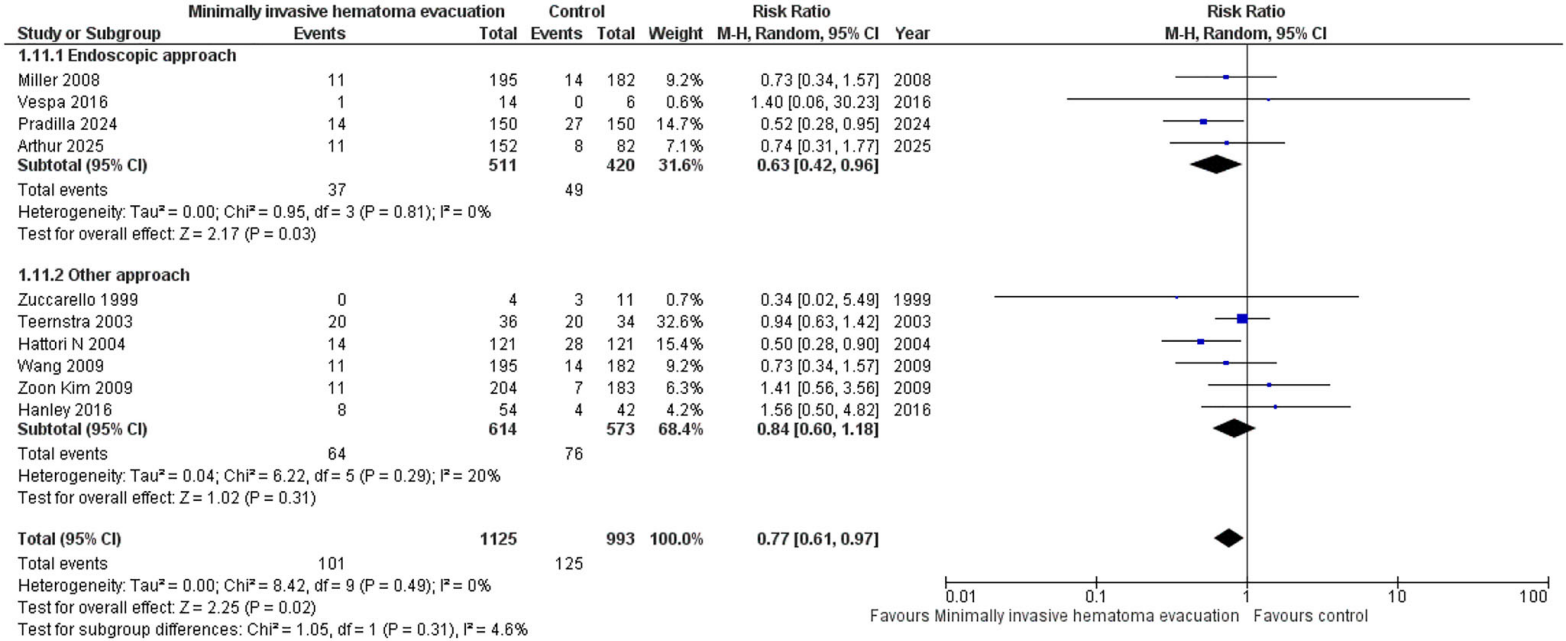
Forest plot showing pooled risk ratios for all‐cause mortality.

In subgroup analysis, the endoscopic group showed a significant trend towards all‐cause mortality in the minimally invasive hematoma evacuation group with a pooled risk ratio of RR = 0.63 (95% CI 0.42 to 0.96; *Z* = 2.17, *p* = 0.03) with no heterogeneity. In contrast, other approach groups showed a nonsignificant trend towards all‐cause mortality for the minimally invasive group with a pooled risk of RR = 0.84, (95% CI 0.60 to 1.18; *Z* = 1.02, *p* = 0.31). Low heterogeneity was observed (*I* ^2^ = 20%). The test for subgroup differences was not significant, with *p* = 0.31 and with low heterogeneity

#### 3.5.2 Mortality at Six Months

6 studies, including 1468 patients (787 minimally invasive hematoma evacuation vs. 681 control). The pooled random‐effects model demonstrated a statistically significant difference in six‐month mortality between minimally invasive hematoma evacuation and control, with a pooled risk ratio (RR = 0.79, 95% CI 0.64 to 0.96; *Z* = 2.33, *p* = 0.02). No heterogeneity was observed. As shown in Figure 3, minimally invasive hematoma evacuation was associated with improved survival at six months.

**FIGURE 3:**
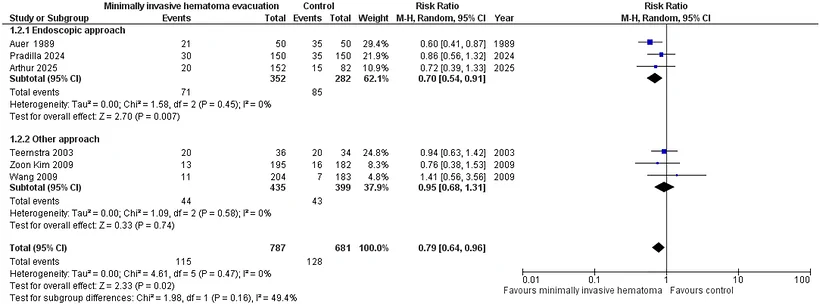
Forest plot showing pooled risk ratios for six‐month mortality.

In subgroup analysis, the endoscopic group showed a significant trend towards mortality at six months in the minimally invasive hematoma evacuation group, with a pooled risk ratio of RR = 0.70 (95% CI 0.54 to 0.91; *Z* = 2.70, *p* = 0.007) and no heterogeneity. In contrast, other approaches showed a non‐significant trend towards all‐cause mortality for the minimally invasive group with a pooled risk of RR = 0.95, 95% CI 0.68 to 1.31; *Z* = 0.33, *p* = 0.74). No heterogeneity was observed

The test for subgroup differences was not significant, with *p* = 0.16 and moderate heterogeneity.

#### 3.5.3 Mortality at 7 Days

Three studies involving a total of 350 patients (220 patients who underwent minimally invasive hematoma evacuation vs. 130 controls) reported 7‐day mortality. The pooled analysis showed no statistically significant difference between the two treatment modalities (RR = 1.02, 95% CI 0.13 to 8.15; *Z* = 0.01, *p* = 0.99). No heterogeneity was noted across the studies, *I* ^2^ = 0%. As shown in Figure 4, there was no significant difference in early mortality between the groups.

**FIGURE 4:**
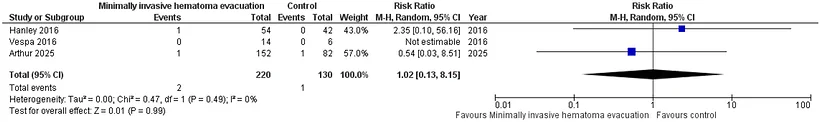
Forest plot showing pooled risk ratios for 7‐day mortality.

#### 3.5.4 Mortality at 30 Days

7 studies, including 1423 patients (774 minimally invasive hematoma evacuation vs 649 control). The pooled random effect model demonstrated a statistically nonsignificant difference in mortality at 30 days for minimally invasive hematoma evacuation over control, with a pooled risk ratio (RR = 0.70, 95% CI 0.49 to 1.01; *Z* = 1.88, *p* = 0.06). No heterogeneity was observed; *I* ^2^ = 0%. As shown in Figure 5, there was no significant difference in mortality at 30 days between the two groups.

**FIGURE 5:**
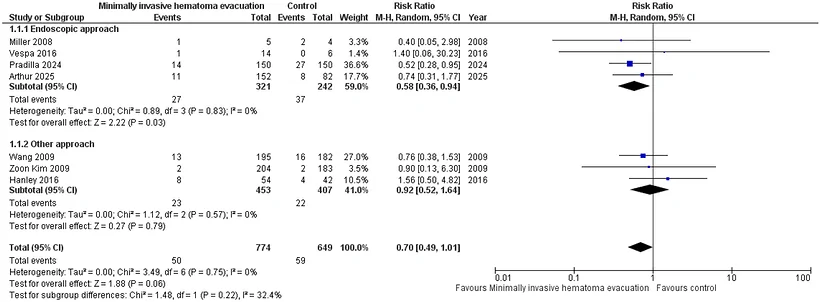
Forest plot showing pooled risk ratios for 30‐day mortality.

In subgroup analysis, the endoscopic group showed a significant trend towards mortality at 30 days in the minimally invasive hematoma evacuation group with a pooled risk ratio of RR = 0.58 (95% CI 0.36 to 0.94; *Z* = 2.22, *p* = 0.03) with no heterogeneity. In contrast, the other approaches group showed a non‐significant trend towards Mortality30 days for the minimally invasive group with a pooled risk of (RR = 0.92, 95% CI 0.52 to 1.64; *Z* = 0.27, *p* = 0.79). No heterogeneity was observed. The test for subgroup differences was not significant, with Moderate heterogeneity of *I* ^2^ = 32.4%.

### 3.6 Meta‐Analysis of Secondary Outcomes

#### 3.6.1 MRS Score of 0–1

Three studies involving a total of 553 patients (291 minimally invasive hematoma evacuation vs 262 control) reported favorable functional outcomes (mRS 0–1). The pooled analysis showed no statistically significant difference between the two treatment modalities (RR = 1.01, 95% CI 0.42 to 2.44; *Z* = 0.02, *p* = 0.98). No heterogeneity was noted across the studies, *I* ^2^ = 0%. As shown in Figure 6, there was no significant difference in favorable functional outcomes between the groups.

**FIGURE 6:**
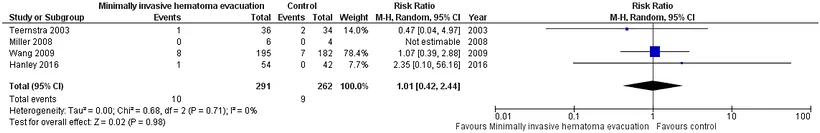
Forest plot showing pooled risk ratios for mRS 0–1.

#### 3.6.2 MRS Score of 0–3 at 180 Days

Four studies involving a total of 628 patients (359 minimally invasive hematoma evacuation vs. 269 control) reported favorable functional outcomes (mRS 0–3) at 180 days. The pooled analysis showed no statistically significant difference between the two treatment modalities (RR = 1.14, 95% CI 0.93 to 1.38; *Z* = 1.27, *p* = 0.20). Low heterogeneity was noted across the studies, *I* ^2^ = 0%. As shown in Figure 7, there was no significant difference in functional outcomes at 180 days between the groups.

**FIGURE 7:**
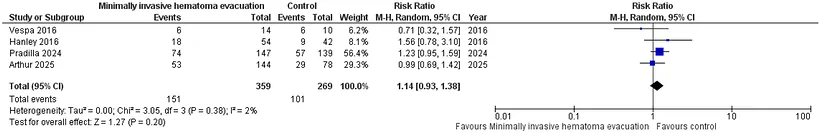
Forest plot showing pooled risk ratios for mRS 0–3 at 180 days.

#### 3.6.3 MRS Score of 0–6

Two studies involving a total of 146 patients (68 minimally invasive hematoma evacuation vs. 78 control) reported mRS scores of 0–6. The pooled analysis showed no statistically significant difference between the two treatment modalities (RR = 0.89, 95% CI 0.62 to 1.27; *Z* = 0.64, *p* = 0.52). High heterogeneity was noted across the studies, *I* ^2^ = 90%. As shown in Figure 8, there was no significant difference in overall functional outcomes between the groups.

**FIGURE 8:**
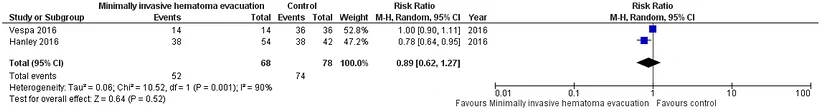
Forest plot showing pooled risk ratios for mRS 0–6.

#### 3.6.4 Mean Modified Rankin Scale (mRS) Score at 180 Days

Two studies involving a total of 777 patients (440 minimally invasive hematoma evacuation vs 337 controls) reported the mean mRS score at 180 days. The pooled analysis showed no statistically significant difference between the two treatment modalities (MD = −0.60, 95% CI −1.87 to 0.67; *Z* = 0.92, *p* = 0.36). As shown in Figure 9, there was no significant difference in mean functional outcome at 180 days between the groups.

**FIGURE 9:**
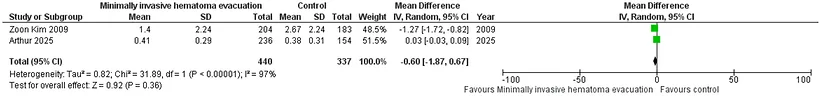
Forest plot showing mean difference in mRS score at 180 days.

#### 3.6.5 Hematoma Volume Reduction

Three studies involving a total of 632 patients (363 minimally invasive hematoma evacuation vs 269 control) reported hematoma volume reduction. The pooled analysis showed a statistically significant difference between the two treatment modalities (MD = 62.47, 95% CI 2.04 to 122.91; Z = 2.03, p = 0.04). High heterogeneity was noted across the studies, *I* ^2^ = 99%. As shown in Figure 10, minimally invasive hematoma evacuation resulted in greater hematoma volume reduction compared to control.

**FIGURE 10:**
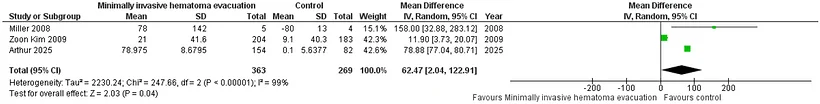
Forest plot showing mean difference in hematoma volume reduction.

#### 3.6.6 Ischemic Stroke

Two studies involving a total of 370 patients (186 minimally invasive hematoma evacuation vs 184 control) reported ischemic stroke. The pooled analysis showed no statistically significant difference between the two treatment modalities (RR = 0.40, 95% CI 0.09 to 1.72; *Z* = 1.24, *p* = 0.22). No heterogeneity was noted across the studies, *I* ^2^ = 0%. As shown in Figure 11, there was no significant difference in the risk of ischemic stroke between the groups.

**FIGURE 11:**
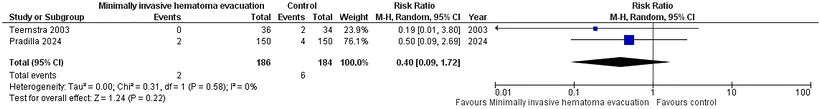
Forest plot showing pooled risk ratios for ischemic stroke.

#### 3.6.7 Length of Hospital Stay

Three studies involving a total of 606 patients (340 minimally invasive hematoma evacuation vs 266 control) reported the length of hospital stay. The pooled analysis showed no statistically significant difference between the two treatment modalities (MD = −0.99, 95% CI −4.13 to 2.16; *Z* = 0.62, *p* = 0.54. High heterogeneity was noted across the studies, *I* ^2^ = 70%. As shown in Figure 12, there was no significant difference in length of hospital stay between the groups

**FIGURE 12:**
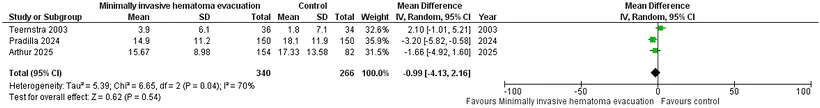
Forest plot showing mean difference in length of hospital stay.

#### 3.6.8 Length of ICU Stay

Three studies involving a total of 606 patients (340 minimally invasive hematoma evacuation vs 266 control) reported the length of ICU stay. The pooled analysis showed no statistically significant difference between the two treatment modalities (MD = −1.05, 95% CI −3.34 to 1.23; *Z* = 0.90, *p* = 0.37). High heterogeneity was noted across the studies, *I* ^2^ = 74%. As shown in Figure 13, there was no significant difference in ICU stay between the groups.

**FIGURE 13:**
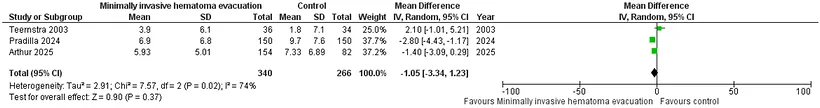
Forest plot showing mean difference in length of ICU stay.

#### 3.6.9 Cardiac Events

Three studies involving a total of 747 patients (381 minimally invasive hematoma evacuation vs 366 control) reported cardiac events. The pooled analysis demonstrated a statistically significant difference between the two groups, with a higher risk observed in the intervention group (RR = 5.08, 95% CI 1.49 to 17.32; *Z* = 2.60, *p* = 0.009). No heterogeneity was noted across the studies, *I* ^2^ = 0%. As shown in Figure 14, minimally invasive hematoma evacuation was associated with an increased risk of cardiac events.

**FIGURE 14:**
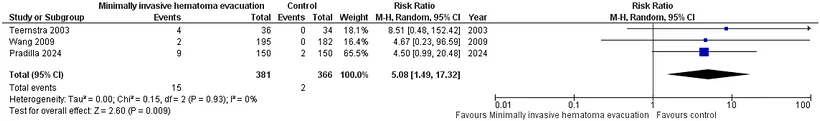
Forest plot showing pooled risk ratios for cardiac events.

#### 3.6.10 Gastrointestinal Bleeding

Three studies involving a total of 755 patients (384 minimally invasive hematoma evacuation vs 371 control) reported GI bleeding. The pooled analysis showed no statistically significant difference between the two treatment modalities (RR = 0.87, 95% CI 0.36 to 2.13; *Z* = 0.30, *p* = 0.77). Moderate heterogeneity was noted across the studies, *I* ^2^ = 37%. As shown in Figure 15, there was no significant difference in gastrointestinal bleeding between the groups

**FIGURE 15:**
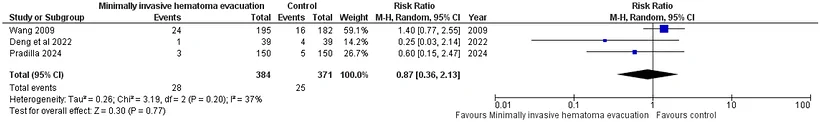
Forest plot showing pooled risk ratios for gastrointestinal bleeding.

#### 3.6.11 Rebleeding

6 studies, including 894 patients (446 minimally invasive hematoma evacuation vs. 448 control). The pooled random effect model demonstrated a statistically significant difference in rebleeding for minimally invasive hematoma evacuation over control with a pooled risk ratio (RR = 2.37, 95% CI 1.37 to 4.10; *Z* = 3.07, *p* = 0.002). No heterogeneity was observed, *I* ^2^ = 0%.As shown in Figure 16, minimally invasive hematoma evacuation was associated with a higher risk of rebleeding.

**FIGURE 16:**
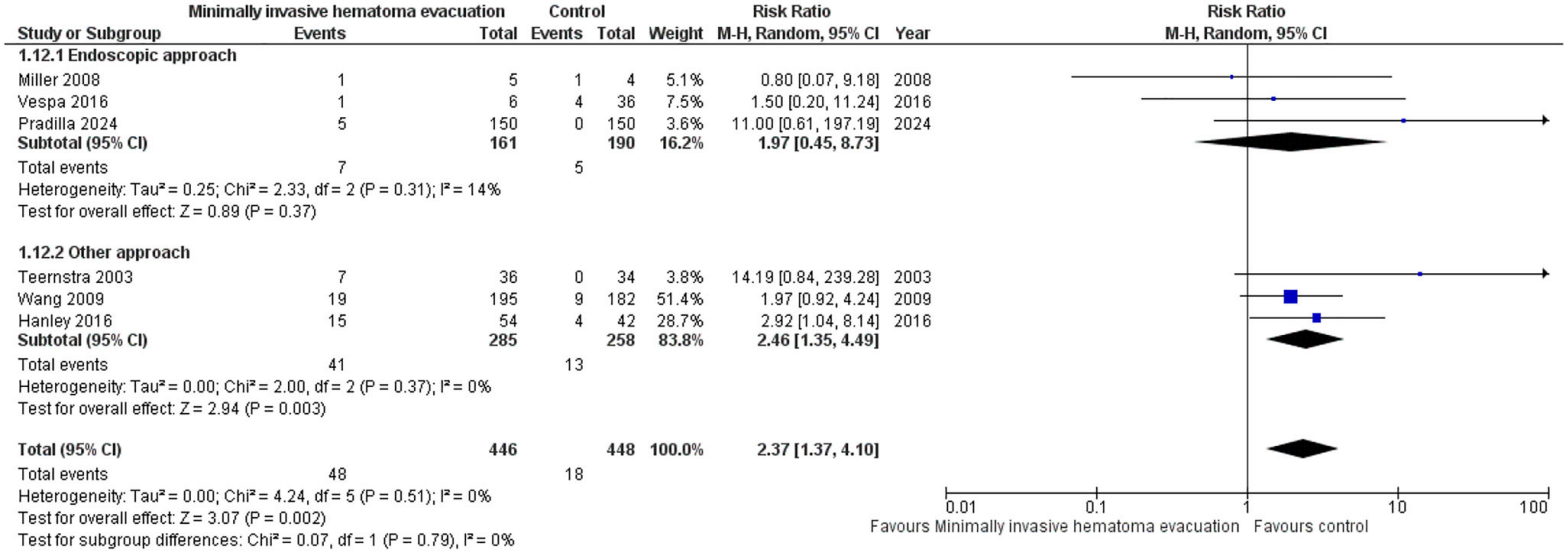
Forest plot showing pooled risk ratios for rebleeding.

In subgroup analysis, the endoscopic group showed a significant trend towards Rebleeding in the minimally invasive hematoma evacuation group, with a pooled risk ratio of (RR = 1.97, 95% CI 0.45 to 8.73; *Z* = 0.89, *p* = 0.37), with moderate heterogeneity of *I* ^2^ = 14%. In contrast, the other approaches group showed a significant trend towards rebleeding for the minimally invasive group with a pooled risk of (RR = 2.46, 95% CI 1.35 to 4.49; *Z* = 2.94, *p* = 0.003). No heterogeneity was observed. The test for subgroup differences was not significant, with *p* = 0.79, and there was no heterogeneity

#### 3.6.12 Renal Failure

Two studies involving a total of 677 patients (345 minimally invasive hematoma evacuation vs 332 control) reported renal failure. The pooled analysis showed no statistically significant difference between the two treatment modalities (RR = 0.60, 95% CI 0.09 to 3.94; *Z* = 0.53, *p* = 0.60). Low heterogeneity was noted across the studies, *I* ^2^ = 27%. As shown in Figure 17, there was no significant difference in the risk of renal failure between the groups.

**FIGURE 17:**
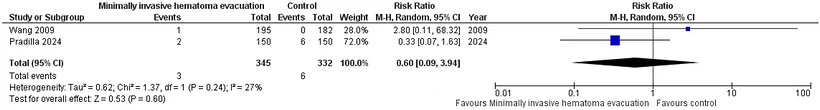
Forest plot showing pooled risk ratios for renal failure.

#### 3.6.13 Serious Adverse Events

Three studies involving a total of 911 patients (497 minimally invasive hematoma evacuation vs 414 control) reported serious adverse events (SAE). The pooled analysis showed no statistically significant difference between the two treatment modalities (RR = 1.14, 95% CI 0.72 to 1.80; *Z* = 0.56, *p* = 0.58). High heterogeneity was noted across the studies, *I* ^2^ = 90% (Figure 18)

**FIGURE 18:**
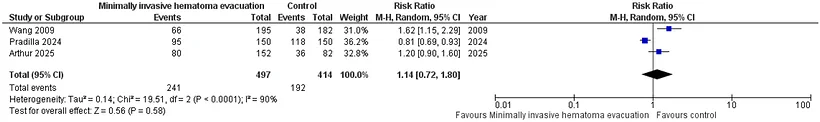
Forest plot showing pooled risk ratios for serious adverse events.

#### 3.6.14 Seizures

Two studies involving a total of 370 patients (186 minimally invasive hematoma evacuation vs 184 control) reported the incidence of seizures. The pooled analysis showed no statistically significant difference between the two treatment modalities (RR = 0.55, 95% CI 0.26 to 1.16; *Z* = 1.57, *p* = 0.12). No heterogeneity was noted across the studies, *I* ^2^ = 0%. As shown in Figure 19, there was no significant difference in seizure incidence between the groups.

**FIGURE 19:**
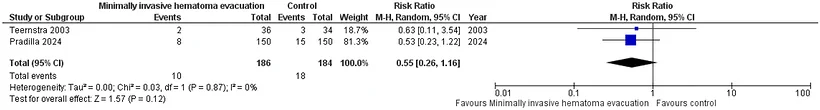
Forest plot showing pooled risk ratios for seizures.

#### 3.6.15 Urinary Tract Infection (UTI)

Three studies involving a total of 448 patients (225 minimally invasive hematoma evacuation vs 223 control) reported urinary tract infections (UTI). The pooled analysis showed no statistically significant difference between the two treatment modalities (RR = 0.64, 95% CI 0.36 to 1.16; *Z* = 1.46, *p* = 0.15). No heterogeneity was noted across the studies, *I* ^2^ = 0%. As shown in Figure 20, there was no significant difference in seizure incidence between the groups.

**FIGURE 20:**
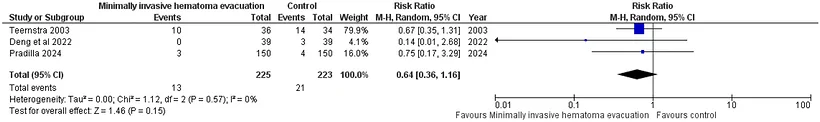
Forest plot showing pooled risk ratios for urinary tract infections.

#### 3.6.16 Trial Sequential Analysis Results

TSA was performed for the primary efficacy outcomes.

### 3.7 All‐Cause Mortality

TSA showed that the cumulative Z‐curve crossed the conventional significance boundary but did not reach the trial sequential monitoring boundary or the RIS (5372 vs. 2118 participants). As shown in Figure 20, this indicates that the apparent reduction in all‐cause mortality with minimally invasive hematoma evacuation is not yet conclusive and warrants further adequately powered trials. (Figure 21)

**FIGURE 21:**
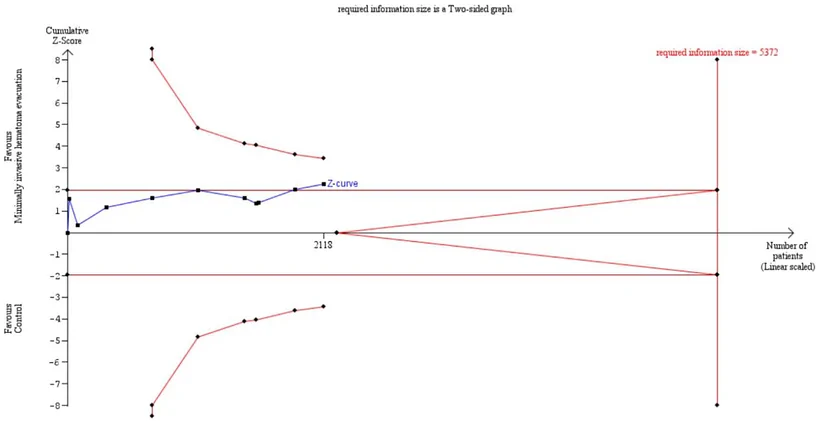
Trial sequential analysis of all‐cause mortality.

### 3.8 6‐Months Mortality

TSA for 6‐month mortality demonstrated that the cumulative Z‐curve crossed the conventional boundary for statistical significance. However, the Z‐curve did not cross the trial sequential monitoring boundary and the accrued information size of 1485 participants remained below the RIS of 2833 participants. These findings suggest that the current evidence is insufficient to confirm a definitive reduction in 6‐month mortality. (Figure 22)

**FIGURE 22:**
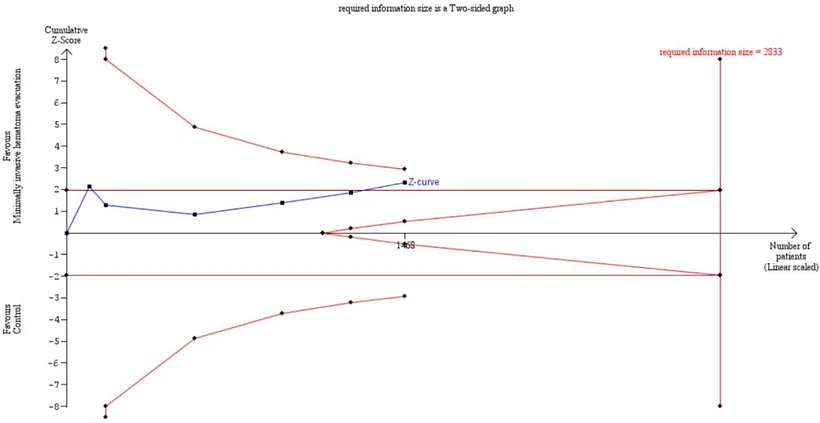
Trial sequential analysis of 6‐month mortality.

### 3.9 7‐Day Mortality

For 7‐day mortality, TSA could not estimate the RIS due to the very low number of events. Consequently, TSA was inconclusive for this outcome, and no firm conclusions can be drawn.

### 3.10 30‐Day Mortality

TSA for 30‐day mortality showed that the cumulative Z‐curve approached and marginally crossed the conventional boundary for statistical significance. However, the cumulative Z‐curve did not cross the trial sequential monitoring boundary and the accrued information size of 1483 participants remained substantially below the RIS of 3043 participants, representing approximately 48.7% of the required sample size. These findings indicate that the available evidence is insufficient to confirm a definitive reduction in 30‐day mortality and remains vulnerable to random errors. (Figure 23)

**FIGURE 23:**
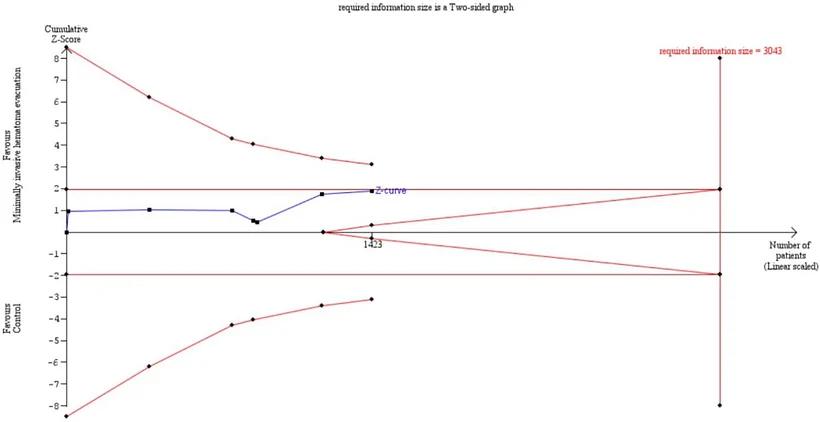
Trial sequential analysis of 30‐day mortality.

## 4 Discussion

Minimally invasive hematoma evacuation in adults with spontaneous supratentorial ICH was associated with a statistically significant reduction in overall mortality compared with best medical management, with a pooled risk ratio ([RR] of 0.77 [95% CI 0.61–0.97] *p* = 0.02) (Alkhiri et al. 2024). Although no clear benefit was seen in very early mortality at 7 days (RR 1.02, 95% CI 0.13–8.15, *p* = 0.99) (Zuccarello et al. 1999; Teernstra et al. 2003), there was a trend toward reduced 30‐day mortality (RR 0.70, 95% CI 0.49–1.01, *p* = 0.06) (De Rosa et al. 2023) and a significant reduction at 6 months (RR 0.79, 95% CI 0.64–0.96, *p* = 0.02) (Alkhiri et al. 2024). These findings reflect a pattern also reported in recent pooled analyses, where longer‐term survival gains emerged despite neutral early outcomes (Alkhiri et al. 2024; Greenberg et al. 2022).

Early randomized trials furnished mixed signals regarding mortality. Pooled data from 10 RCTs demonstrated that minimally invasive hematoma evacuation significantly reduced all‐cause mortality (RR 0.77, 95% CI 0.61–0.97, *p* = 0.02) (Alkhiri et al. 2024). This finding is consistent with the long‐term survival advantage reported in the MISTIE III trial, where Hanley et al. observed improved functional outcomes and survival associated with effective clot reduction, although early mortality did not reach significance (Hanley et al. 2019). Earlier RCTs, including Zuccarello et al. (Zuccarello et al. 1999), Teernstra et al. (Teernstra et al. 2003), Hattori et al. (Hattori et al. 2004), Miller et al. (Miller et al. 2008), Wang et al. (Wang et al. 2009), and Zoon Kim et al. (Kim and Kim 2009), were underpowered to detect mortality differences, reinforcing the importance of pooling data in meta‐analyses to identify a survival signal. The pathophysiology of ICH involves initial hematoma formation followed by secondary brain injury, including the release of hemoglobin and other toxic blood breakdown products that disrupt the blood–brain barrier, cause oxidative stress, and promote neuroinflammation (Wang et al. 2016; Zhang et al. 2022; Chen et al. 2022). Elevated ICP from hematoma mass and perihematomal edema leads to cerebral perfusion compromise and herniation if unchecked (Marchina et al. 2022; Selim and Norton 2020; Qureshi et al. 2001). Minimally invasive evacuation directly removes the mass lesion, reduces pressure, and may limit these neurotoxic cascades, thereby lowering the risk of death.

Early mortality at 7 days did not differ significantly between MIS and medical management (RR 1.02, 95% CI 0.13–8.15) *p* = 0.99) (Zuccarello et al. 1999; Teernstra et al. 2003). This aligns with previous observations that early deaths in ICH are often related to initial hematoma size, location, and baseline comorbidities rather than the surgical intervention itself (Hanley et al. 2019). Smaller early trials generally reported neutral effects on short‐term mortality (Zuccarello et al. 1999; Teernstra et al. 2003; Pradilla et al. 2024), emphasizing that minimally invasive evacuation may not immediately alter hyperacute risk but has delayed benefits as the mass effect resolves and secondary injury pathways are mitigated.

At 30 days, there was a trend toward reduced mortality with minimally invasive surgery (RR 0.70, 95% CI 0.49–1.01, *p* = 0.06) (Pradilla et al. 2024). This near‐significant reduction is supported by findings from the ENRICH trial, which reported 30‐day mortality of 9.3% in the surgical arm versus 18.0% in controls (Pradilla et al. 2024). The pattern suggests that surgical removal of the hematoma may require time to translate into survival benefit, likely reflecting progressive reduction in ICP, perihematomal edema, and secondary neuronal injury over the first few weeks post‐intervention.

Six‐month mortality was significantly lower in patients undergoing minimally invasive evacuation (RR 0.79, 95% CI 0.64–0.96, *p* = 0.02) (Alkhiri et al. 2024). This reinforces the long‐term survival advantage noted in prior meta‐analyses (Yao et al. 2018; Hallenberger et al. 2022) and highlights the durability of the intervention's effect. These findings suggest that although short‐term mortality remains largely unaffected, the cumulative impact of clot reduction, mitigation of secondary injury, and optimization of cerebral perfusion may translate into improved long‐term outcomes.

Secondary outcomes unveil a complex risk‐benefit profile. Hematoma volume reduction was substantially greater with minimally invasive evacuation (MD 62.47 mL, 95% CI 2.04–122.91, *p* = 0.04) (Vespa et al. 2016), corroborating findings from ICES and other endoscopic series demonstrating rapid clot burden diminution with MIS techniques. However, this radiological advantage was accompanied by significantly higher rates of rebleeding (RR 2.37, 95% CI 1.37–4.10, *p* = 0.002) (Higgins et al. 2024; Sterne et al. 2019), echoing the increased bleeding risk seen when surgical manipulation intersects fragile perihematomal vasculature. Cardiovascular events were also more frequent (RR 5.08, 95% CI 1.49–17.32, *p* = 0.009) (Li et al. 2020), an association potentially related to the neuroendocrine and inflammatory stress response to surgery, sympathetic activation with catecholamine surges, hemodynamic perturbations, and perioperative fluid shifts that increase myocardial oxygen demand and cardiac workload (Desborough 2000).

Neurological outcomes such as ischemic stroke did not differ significantly between groups (RR 0.40, 95% CI 0.09–1.72, *p* = 0.22) (Sondag et al. 2020), consistent with prior reviews indicating that MIS affects mass effect more than distal perfusion deficits. Bleeding outside the central nervous system, such as gastrointestinal hemorrhage, showed no meaningful difference (RR 0.87, 95% CI 0.36–2.13) *p* = 0.77) (Steiner et al. 2014), suggesting systemic bleeding risk remains more tied to comorbidity profiles than to surgical approach. Seizures, renal failure, and urinary tract infection rates were similarly unaffected, indicating that these complications are driven more by baseline patient factors and critical care management than by the choice of hematoma removal strategy.

Heterogeneity was low for primary mortality outcomes, with *I* ^2^ = 0% across all measured time points, which strengthens confidence in the consistency of the survival benefit. However, measures like length of ICU stay (*I* ^2^ = 74%), length of hospital stay (*I* ^2^ = 70%), and SAEs (*I* ^2^ = 90%) demonstrated high heterogeneity, likely reflecting differences in institutional care pathways, definitions of adverse events, and reporting frameworks across trials conducted over two decades. Furthermore, the analyses were based on aggregate study‐level data and univariate pooling of reported outcomes. Consequently, important clinical variables such as baseline neurological severity, hematoma characteristics, comorbidities, perioperative complications, and institutional management practices could not be adjusted for in the pooled analyses. This limitation is particularly relevant for outcomes such as ICU stay and hospital stay, which are influenced by multiple patient‐ and treatment‐related factors. Therefore, these findings should be interpreted as unadjusted associations rather than independent effects of minimally invasive hematoma evacuation.

The biological rationale supporting these clinical effects centers on the mitigation of mass effect and secondary injury mechanisms. Removing the clot reduces ICP and the production of neurotoxic blood breakdown products known to propagate perihematomal edema and neuronal apoptosis (Greenberg et al. 2022). Improved clearance may curtail inflammatory cascades and facilitate earlier restoration of cerebral autoregulation, which can translate into improved long‐term survival and functional outcomes. Notably, extensive clot evacuation appears critical, as partial removal strategies without significant residual reduction have not consistently translated to improved outcomes in prior trials (Hanley et al. 2019).

### 4.1 Strengths

This meta‐analysis incorporates several methodological strengths. By restricting inclusion to randomized controlled trials, the analysis minimizes selection bias and maximizes internal validity (Higgins et al. 2022). Low heterogeneity in primary mortality outcomes (*I* ^2^ = 0%) supports consistency across included trials and enhances the reliability of pooled estimates (Alkhiri et al. 2024). Inclusion of both contemporary trials, such as ENRICH (Pradilla et al. 2024), and earlier RCTs, including MISTIE III (Hanley et al. 2019), allows for temporal and technological breadth, capturing improvements in surgical technique and perioperative care. Outcome measures were aligned with established guideline endpoints from the AHA/ASA (Greenberg et al. 2022) and European Stroke Organization (Steiner et al. 2014), ensuring clinical relevance. Additionally, this meta‐analysis evaluates both short‐term and long‐term mortality alongside secondary neurological, radiological, and systemic outcomes, providing a comprehensive risk‐benefit assessment. Our meta‐analysis adds to the existing literature in several important ways. Unlike prior meta‐analyses, which often combined heterogeneous populations, including secondary or infratentorial ICH or mixed surgical approaches (Yao et al. 2018; Hallenberger et al. 2022), our study focuses exclusively on spontaneous supratentorial ICH and restricts inclusion to randomized controlled trials. This allows for more precise estimation of mortality benefits and secondary outcomes specific to this population.

Our meta‐analysis contributes to the existing literature by providing a focused evaluation of minimally invasive surgery in spontaneous supratentorial ICH. While recent evidence syntheses, including the Cochrane review by Wilting et al., have assessed a broad spectrum of surgical interventions for ICH, our study specifically examines minimally invasive hematoma evacuation techniques and restricts inclusion to randomized controlled trials. This focused approach provides a dedicated assessment of the efficacy and safety of MIS within a clinically relevant patient population. Furthermore, by evaluating contemporary and historical randomized evidence, our study offers an updated synthesis of MIS‐specific outcomes, including mortality, functional recovery, radiological outcomes, and safety endpoints.

### 4.2 Limitations

Despite these strengths, the analysis has important limitations. Clinical and methodological heterogeneity persists for secondary outcomes, including ICU stay, hospital stay, and SAEs, likely due to variable perioperative protocols, reporting standards, and operator expertise (Sondag et al. 2020; Hallenberger et al. 2022). Many included RCTs were small and underpowered for rare outcomes such as cardiac events and procedural complications, limiting precision (Li et al. 2020). Variation in surgical modalities, including endoscopic, stereotactic, and craniopuncture techniques introduce potential confounding due to differences in procedural efficacy and complication profiles (Vespa et al. 2016; Yao et al. 2018). Inconsistencies in the timing of intervention, patient selection criteria, and post‐operative care pathways may have contributed to residual bias and outcome variability

Although subgroup analyses were performed where feasible, several subgroups contained relatively small numbers of studies and participants, limiting statistical power and the ability to fully explore sources of heterogeneity. Furthermore, only a limited number of included trials were assessed as having a low RoB, which may affect the certainty of the available evidence. The inclusion of several small randomized trials also limits precision for infrequent outcomes such as cardiac events and procedural complications. Importantly, while conventional meta‐analysis demonstrated a reduction in mortality associated with MIS, TSA indicated that the RIS has not yet been reached. Therefore, the current evidence should be considered suggestive rather than definitive, and additional adequately powered randomized trials are needed to confirm the observed benefits. Another limitation is the use of the DerSimonian and Laird random‐effects model for all pooled analyses. Although this approach is widely used in meta‐analytic research, it may underestimate between‐study variance and produce narrower confidence intervals when the number of included studies is small. Consequently, the precision of some pooled estimates should be interpreted with caution, and future analyses may benefit from the application of more robust variance estimation methods, such as restricted maximum likelihood or Hartung–Knapp adjustments. Additionally, the assessment of functional outcomes was limited by variability in outcome reporting across studies. The included trials reported mRS outcomes using different definitions and thresholds, including mRS 0–1, mRS 0–3, and mean mRS scores. Because individual patient‐level data were unavailable, it was not possible to apply a uniform functional outcome definition or perform an ordinal shift analysis across all studies. To avoid introducing bias, outcomes were pooled according to the definitions reported in the original trials without deriving new cut‐offs or reclassifying patients. Nevertheless, this heterogeneity in functional outcome reporting may limit the comparability and interpretability of the pooled analyses.

Future studies should focus on identifying the “sweet spot” for intervention timing and patient selection using advanced neuroimaging. Investigating whether specific MIS techniques (e.g., the use of the Apollo system or specific endoscopes) yield superior results compared to others is necessary. Furthermore, upcoming trials such as ENRICH and MIND should provide more definitive data on whether the mortality benefit seen here consistently translates into improved functional quality of life.

## 5 Conclusion

Minimally invasive hematoma evacuation, particularly endoscopic and stereotactic approaches, was associated with reduced long‐term mortality and greater hematoma volume reduction in adults with spontaneous supratentorial ICH. However, TSA indicated that the available evidence remains insufficient for definitive conclusions regarding mortality benefit. Early mortality and functional outcomes were less consistent across studies. Further adequately powered randomized trials with standardized protocols are needed to confirm these findings and better define the role of minimally invasive surgery in ICH management.

## Author Contributions

Conceptualization and study design: Sahar Saleem, Khushal Khan, Khawaja Arham Jawaid, Abdullah Abdullah. Literature search and screening: Muhammad Abbas, Umer Zaryab Khan, Muhammad Waqas, Zuha Kashif. Data extraction and quality assessment: Humaira Obaid, Zahra Rehman, Zaryab Bacha, Javeria Javed. Statistical analysis and interpretation: Khushal Khan, Muhammad Abbas, Umer Zaryab Khan. Manuscript drafting: Sahar Saleem, Khawaja Arham Jawaid, Muhammad Waqas, Zuha Kashif, Abdullah Abdullah.

## Funding

The authors have nothing to report.

## Ethics Statement

The authors have nothing to report.

## Conflicts of Interest

The authors declare no conflicts of interest.

## Supporting information




**Supplementary Materials**: brb371815‐sup‐0001‐SuppMat.docx

## Data Availability

This published article (and its supplementary files, if any) includes all data generated or analyzed during this study.
