# Supplementary material for: Minimally Invasive Hematoma Evacuation for Acute Spontaneous Supratentorial Intracerebral Hemorrhage: A Systematic Review and Meta‐Analysis With Trial Sequential Analysis
Source: Brain Behav. 2026 Oct 14;16(10):e71815. doi: 10.1002/brb3.71815 (PMC13662624; doi:10.1002/brb3.71815)
Supplement: Supplementary file 1 [file BRB3-16-e71815-s001.docx]

|  | **Outcomes** | **Risk of Bias** | **Inconsistency** | **Indirectness** | **Imprecision** | **Publication Bias** | **Quality** |
| --- | --- | --- | --- | --- | --- | --- | --- |
| 1. | All cause mortality | Not serious | Not Serious | Not serious | Not Serious | N/A | High Quality |
| 2. | 30 day mortality | Not serious | Not serious | Not serious | Not Serious | N/A | High Quality |
| 3. | 7 day mortality | Not serious | Not serious | Not serious | Serious | N/A | Moderate Quality |
| 4. | 6 months mortality | Not serious | Not serious | Not serious | Not Serious | N/A | High Quality |
| 5. | Mrs 0-1 | Not serious | Not serious | Not serious | Serious | N/A | Moderate Quality |
| 6. | Mrs 0-3 | Not serious | Not serious | Not serious | Serious | N/A | Moderate Quality |
| 7. | Mrs 0-6 | Not serious | Serious | Not serious | serious | N/A | Low Quality |
| 8. | Myocardial Infarction | Not serious | Not serious | Not serious | Serious | N/A | Moderate Quality |
| 9. | Rebleeding | Not serious | Not Serious | Not serious | Not Serious | N/A | High Quality |
| 10. | Symp bleeding post 72 hours | Not serious | Serious | Not serious | serious | N/A | Low Quality |
| 11. | Haematoma volume reduction | Not serious | serious | Not serious | Not serious | N/A | Moderate Quality |
| 12. | Ischemic Stroke | Not serious | Not Serious | Not serious | Serious | N/A | Moderate Quality |
| 13. | Cardiac events | Not serious | Not serious | Not serious | Not Serious | N/A | High Quality |
| 14. | GI bleeding | Not serious | Not serious | Not serious | Serious | N/A | Moderate Quality |
| 15. | LOH stay in days | Not serious | serious | Not serious | Serious | N/A | Low Quality |
| 16. | Length of ICU stay in days | Not serious | serious | Not serious | Serious | N/A | Low Quality |
| 17. | Pneumonia | Not serious | Not serious | Not serious | Not Serious | N/A | High Quality |
| 18. | Renal failure | Not serious | Not serious | Not serious | Serious | N/A | Moderate Quality |
| 19. | UTI | Not serious | Not serious | Not serious | Serious | N/A | Moderate Quality |
| 20. | Serous adverse events | Not serious | serious | Not serious | Serious | N/A | Low Quality |

- LOH stay= length of hospital stay in days

Table S1. GRADE assessment of included studies

Figure S1. Quality assessment of included studies
